# Intravital fluorescence microscopy with negative contrast

**DOI:** 10.1371/journal.pone.0255204

**Published:** 2021-08-05

**Authors:** Juwell W. Wu, Yookyung Jung, Shu-Chi A. Yeh, Yongwan Seo, Judith M. Runnels, Christian S. Burns, Toshihide Mizoguchi, Keisuke Ito, Joel A. Spencer, Charles P. Lin

**Affiliations:** 1 Center for Systems Biology and Wellman Center for Photomedicine, Massachusetts General Hospital and Harvard Medical School, Boston, Massachusetts, United States of America; 2 Center for Molecular Spectroscopy and Dynamics, Institute for Basic Science, Seoul, Republic of Korea; 3 Department of Chemical and Biomolecular Engineering, Korea Advanced Institute of Science and Technology, Daejeon, Republic of Korea; 4 Department of Bioengineering, University of California Merced, Merced, California, United States of America; 5 NSF-CREST Center for Cellular and Biomolecular Machines and the Health Science Research Institute, University of California Merced, Merced, California, United States of America; 6 Ruth L. and David S. Gottesman Institute for Stem Cell and Regenerative Medicine Research, Departments of Cell Biology and Medicine, Albert Einstein College of Medicine, Bronx, New York, United States of America; 7 Oral Health Science Center, Tokyo Dental College, Tokyo, Japan; Hungarian Academy of Sciences, HUNGARY

## Abstract

Advances in intravital microscopy (IVM) have enabled the studies of cellular organization and dynamics in the native microenvironment of intact organisms with minimal perturbation. The abilities to track specific cell populations and monitor their interactions have opened up new horizons for visualizing cell biology *in vivo*, yet the success of standard fluorescence cell labeling approaches for IVM comes with a “dark side” in that unlabeled cells are invisible, leaving labeled cells or structures to appear isolated in space, devoid of their surroundings and lacking proper biological context. Here we describe a novel method for “filling in the void” by harnessing the ubiquity of extracellular (interstitial) fluid and its ease of fluorescence labelling by commonly used vascular and lymphatic tracers. We show that during routine labeling of the vasculature and lymphatics for IVM, commonly used fluorescent tracers readily perfuse the interstitial spaces of the bone marrow (BM) and the lymph node (LN), outlining the unlabeled cells and forming negative contrast images that complement standard (positive) cell labeling approaches. The method is simple yet powerful, offering a comprehensive view of the cellular landscape such as cell density and spatial distribution, as well as dynamic processes such as cell motility and transmigration across the vascular endothelium. The extracellular localization of the dye and the interstitial flow provide favorable conditions for prolonged Intravital time lapse imaging with minimal toxicity and photobleaching.

## Introduction

IVM has emerged as a powerful tool for studying cellular organization and dynamics in intact organisms [1, 2]. An outstanding example is the analysis of immune cell migration and interaction in LNs that has revolutionized our understanding of antigen presentation and immune cell activation *in vivo* [3–6]. Advances in IVM have given us the ability to track specific cell populations labeled with fluorescent dyes or fluorescent proteins (FPs). A limitation with specific labeling, however, is that only cells that are tagged are visualized. As a result, labeled cells often appear isolated, when they are in fact neighbored with many other (unlabeled) cells and extracellular matrix components. This limitation can be partially overcome by incorporating other nonlinear imaging modalities [7], increasing the number of imaging channels and labeling multiple cell populations at the expense of increasing complexity. However, the cellular constitution of most tissues is highly diverse, and multiplexed imaging is often restricted by spectral overlap. Here we describe a method that takes advantage of the concept of negative contrast and the ubiquitous presence of extracellular fluids to visualize the unlabeled cell populations in BM and LN (S1a and S1b Fig). The method requires a single fluorescence channel, does not entail any modification to existing microscopes, and can be used in tandem with conventional fluorescent tags.

Negative contrast imaging [8] is based on the concept that an object can be discerned when its background is illuminated, even if no light is emitted or reflected from the object itself. When applied to fluorescence microscopy, individual unlabeled cells are the objects while the immediate surroundings, such as the interstitium, serve as the background (S1b Fig). Negative contrast imaging is particularly useful for imaging tissues such as BM or LN, in which numerous cell types coexist and are difficult to visualize simultaneously using standard labeling approaches.

In this paper, we show that during routine labeling of the vasculature and lymphatics for IVM, commonly used fluorescent tracers also provide negative contrast images of BM and LN by perfusing the interstitial spaces and outlining the unlabeled cells. The fluorescence signals originating from the interstitial spaces are frequently overlooked or discarded as “background” but are in fact useful complements to the standard (positive) cell labels, providing a more comprehensive view of the cellular landscape and dynamic processes such as cell motility, transmigration and cell-cell interaction, all without the need for additional contrast agents.

## Results

### Implementation of negative contrast imaging

We implemented intravital negative contrast imaging by filling the interstitial space with a cell-impermeable fluorescent tracer such as Evans blue and members of the fluorescent dextran family, which provides a fluorescent background to the unlabeled cells when imaged by two-photon microscopy. These tracers are routinely used in IVM to label the vasculature (through intravenous injection) or the lymphatics (through distant site injection) [9–12]. The intravenously delivered tracers readily perfuse the BM interstitium due to the high permeability of the sinusoidal vessels [9]. Meanwhile, the tracers injected subcutaneously into a remote location drains through lymphatic vessels into the LN. In both cases, negative contrast images are generated alongside positive labeling of the target structures (the vasculature and the lymphatics).

**Fig 1:**
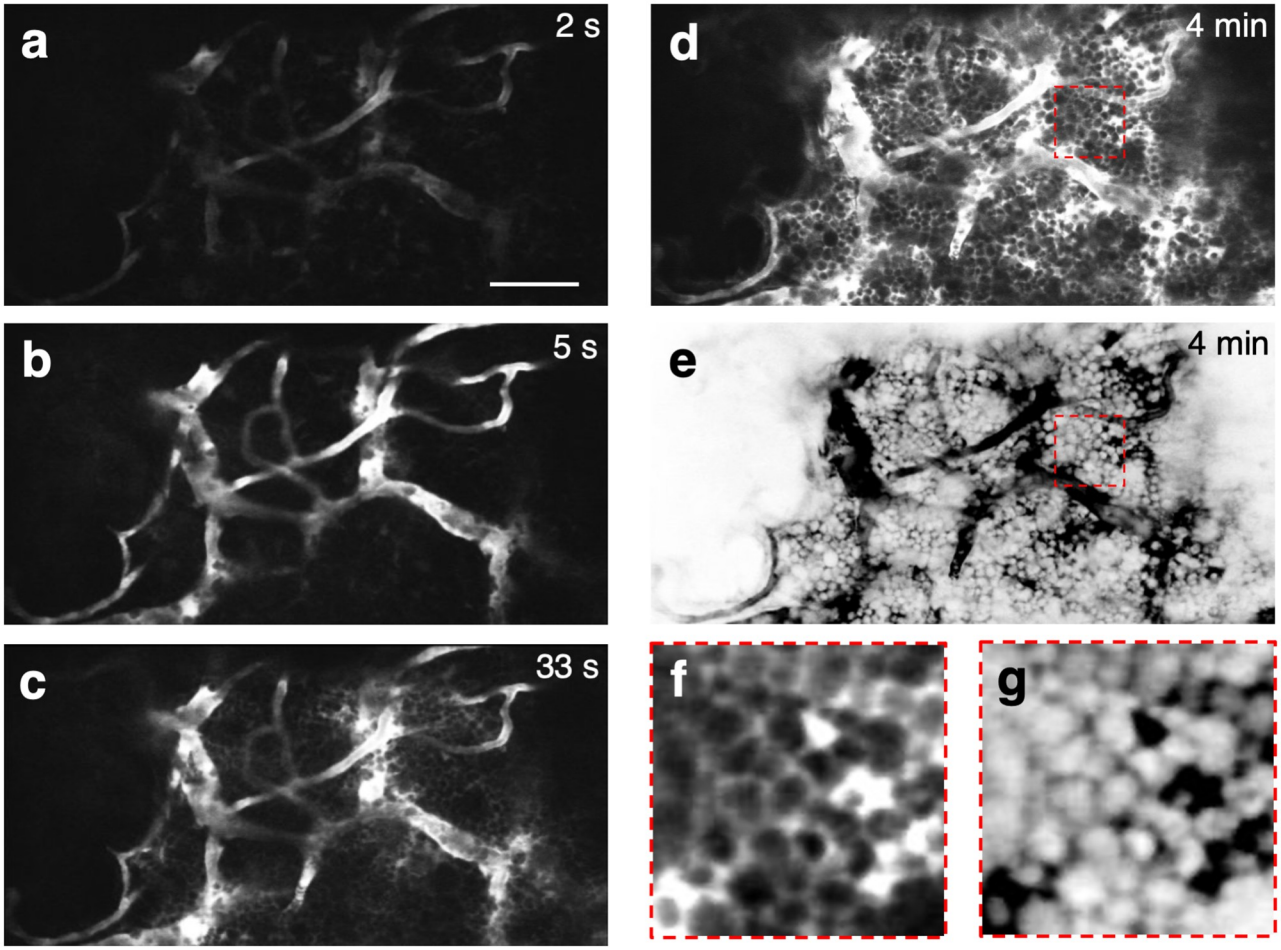
Two-photon negative contrast imaging of a mouse BM cavity, at different time points after the injection of a vascular dye. **a–d**, Images taken at 2 s, 5 s, 33 s and 4 min after tail-vein injection of 70 kDa Texas Red-dextran, respectively. Vascular signal was detected 2–5 s after injection. Leakage of the dye into the extravascular space could be seen by 33 s. By 4 min, the dye occupied the interstitial space between cells, which remained as dark objects in the image. **e**, The LUT of **(d)** was digitally inverted such that cells appeared as bright objects above the dark interstitial background. **f-g**, The dotted areas in **(d,e)** were magnified to show how individual cells appear in negative contrast imaging. Scale bar = 50 μm. Representative example from N = 3 mice.

Fig 1 shows the negative contrast images of the BM acquired during and at different time points after on-stage intravenous injection of 70 kDa Texas Red-dextran into the mouse tail vein (the real-time movie is shown in S1 Movie). The initial filling of the BM vasculature with the dye can be seen beginning at 2–5 seconds after dye injection (Fig 1a and 1b). Leakage of the dye into the interstitial space begins immediately and can be easily discerned as early as the 33 second time point (Fig 1c). By 4 minutes, the dye has permeated the interstitial space and outlined most of the cells in the BM (Fig 1d). Digitally inverting the image and contrast enhancement result in the appearance of bright cells against a dark background, as shown in the last frame of the sequence (Fig 1e) and the enlarged areas from the images taken at 4 minutes (Fig 1f and 1g). Another example of dye delivery is shown in S2 Fig and S2 Movie. For comparison, we have included a histological section of the mouse long bone showing the high density of cells in the marrow (S3 Fig).

**Fig 2:**
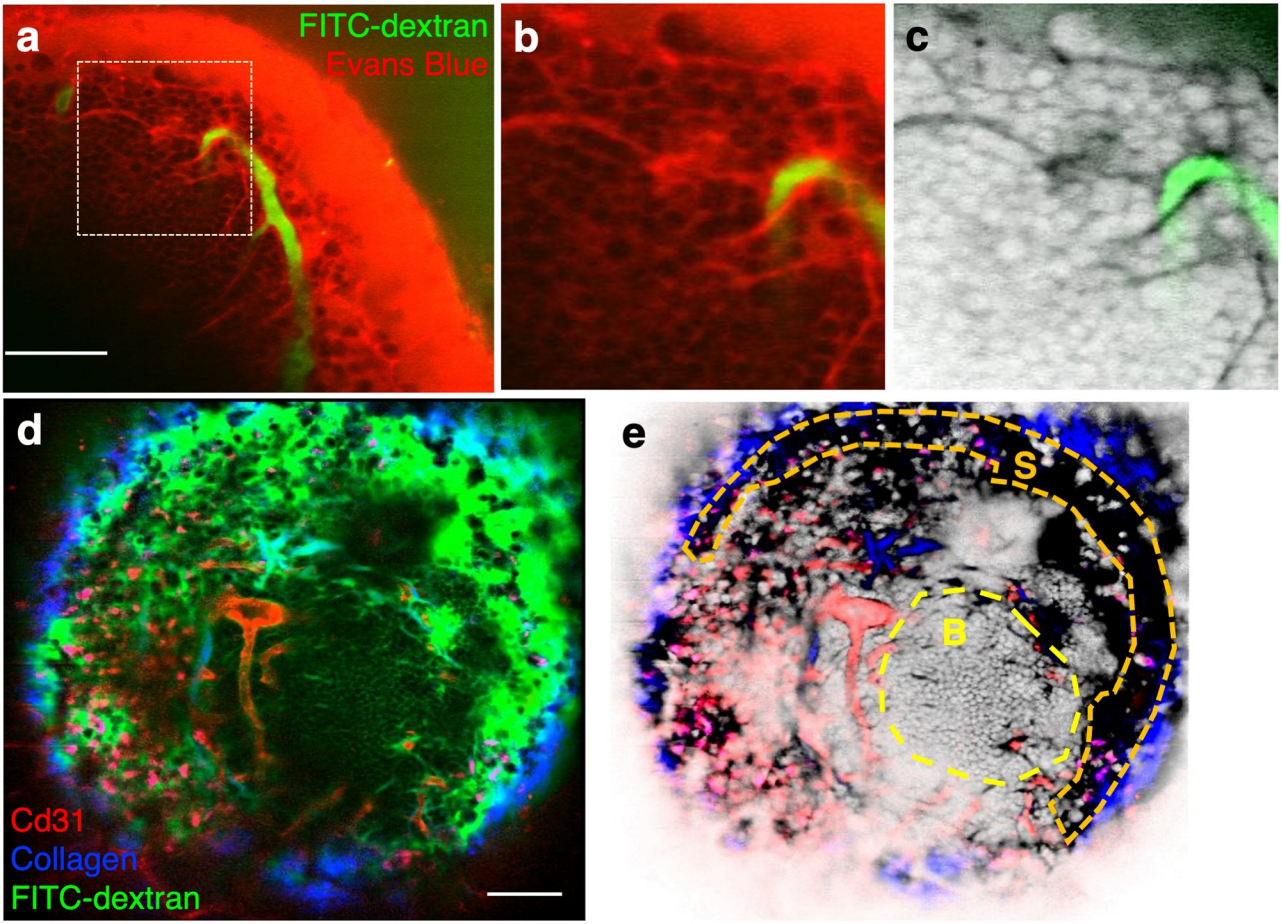
Two-photon negative contrast imaging of the popliteal lymph node. **a**, Interstitium labeling via lymphatic drainage. Evans blue (red) was delivered at a distant site (footpad) and into the popliteal LN via the lymphatic system. FITC-dextran (green) was injected intravenously to visualize the blood vessels. **b**, Zoomed-in view of the dotted region in **(a)**, showing dark individual cells above the fluorescent interstitial background. Small vessel-like structures are lymphatic conduits that branch from the subcapsular sinus. **c**, Same as **(b)**, after LUT inversion. N = 1 mouse. **d**, Popliteal lymph node imaged by a combination of negative contrast imaging of the follicular B cells, positive contrast imaging of the lymphatic vessels and vasculature, and second harmonic generation imaging of the LN capsule. FITC-dextran (green) was injected into footpad for lymphatic vessels and interstitium labeling. Anti CD31-Alexa 594 (red) was injected intravenously for vascular labeling. The SHG signal from the capsule is shown in blue. Punctate labeling in red is due to non-specific antibody uptake by macrophages. **e**, Same as **(d)**, but with the green channel‘s LUT inverted and displayed in grayscale. The dotted area designated “S” indicates the position of the lymphatic sinus and the area designated “B”, the B cell follicle. Negative contrast imaging renders the cells in the follicle readily visible. N = 1 mouse. Scale bar for all images = 50 μm.

Fig 2 shows the in vivo negative contrast images of exposed popliteal LNs after subcutaneous injection of Evans blue (Fig 2a–2c) or 70 kDa FITC-dextran (Fig 2d and 2e) into the mouse foot pad. The dye drained through lymphatic vessels and accumulated in the subcapsular sinus (SCS) within minutes after dye injection (Fig 2a). It entered a network of fine conduits originating from the SCS (Fig 2b), previously shown to transport macromolecules with molecular weight below 70 kDa [13, 14], and percolated through the interstitial space of the B cell follicle, thereby permitting negative contrast imaging of the cells in the region (Fig 2c). We also performed LN multimodal imaging with combined positive and negative contrast. In Fig 2d and 2e, the vasculature was labeled with anti-CD31/Alexa 594 (red) and the lymphatic sinuses were labeled with FITC-dextran (green). The collagenous capsule was visualized by second harmonic generation. Follicular B cells were imaged using negative contrast arising from FITC-dextran-perfused follicular interstitium.

### Comparison of negative contrast imaging with standard fluorescence labeling methods

**Fig 3:**
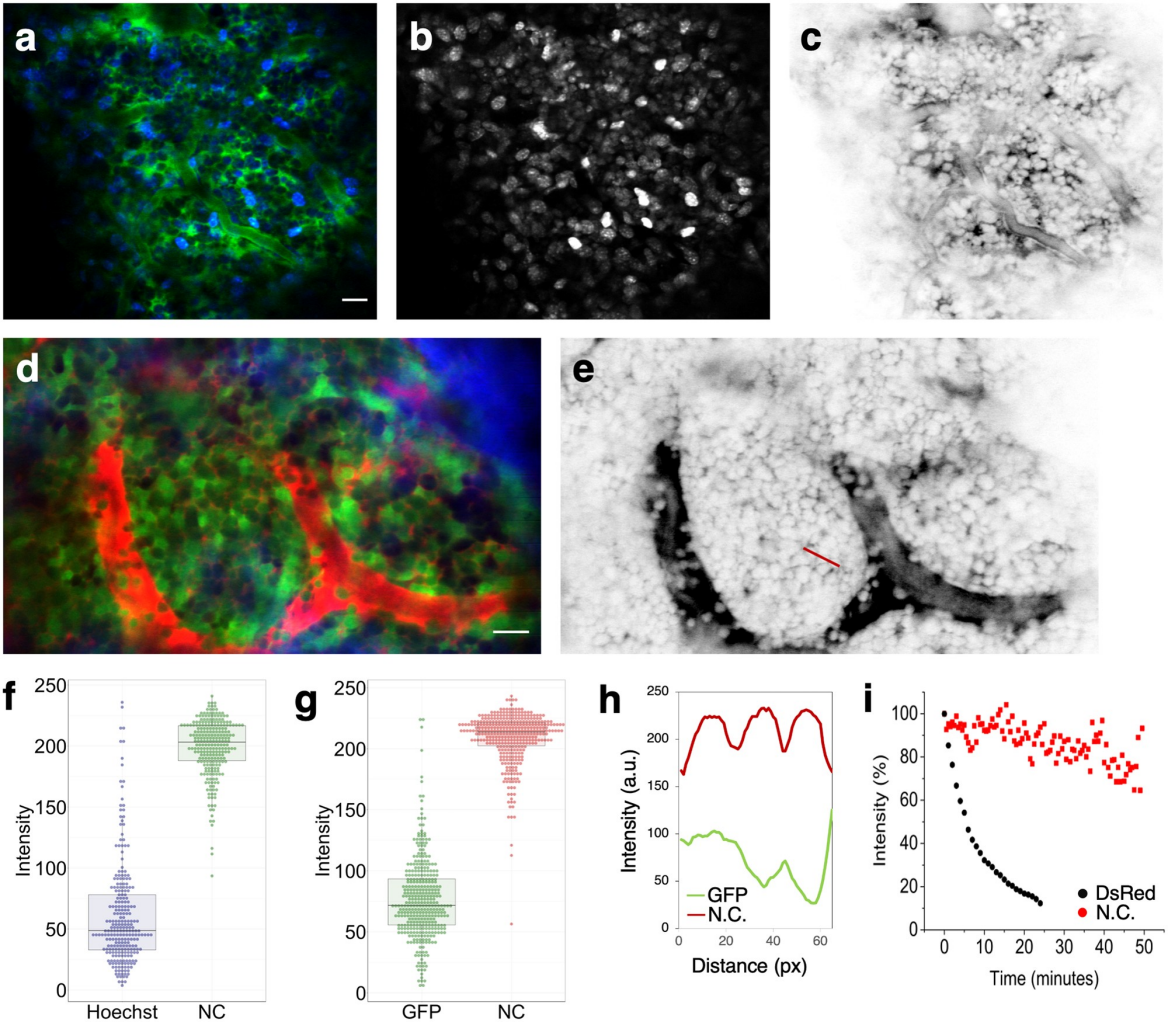
Comparison of negative contrast imaging with standard “universal” fluorescence labeling methods. **a**, Colocalized image of BM cells, labeled with Hoechst 33342 (blue) and interstitial space, labeled with TRITC-dextran (green). **b**, The Hoechst channel shown in gray scale. **c**, The TRITC channel in gray scale after LUT inversion. N = 2 mice. **d**, Colocalized image of BM cells expressing actin-GFP (green) and interstitial space labeled by rhodamine B-dextran (red). Bone second-harmonic generation signal is shown in blue. **e**, The rhodamine B channel in gray scale after LUT inversion. N = 1 mouse with 6 different z-stack locations. **f**, Boxplots of cell intensities in (**a**). 226 cells were included. **g**, Boxplots of cell intensities in (**d**). 369 cells were included. **h**, GFP (green) and negative contrast (red) intensity profiles along the red line marked in (**e**), which spans the width of 3 cells. The negative contrast profile is more consistent than the GFP profile from cell to cell, and the cell boundaries are well-defined. **i**, Photobleaching kinetics of actin-DsRed labeled cells (black) and FITC-dextran labeled interstitium (red). Signal was acquired from BM in vivo, and the excitation laser was continuously scanned over the field of imaging during signal acquisition. N = 2 mice. Scale bar for all images = 20 μm.

In standard immunofluorescence microscopy, counterstains such as DAPI (4′,6 diamidino-2-phenylindole) and Hoechst dyes are frequently used in combination with specific cell labels to “paint” the cellular landscape surrounding the labeled cells. Hoechst 33342 is a nuclear counterstain that is compatible with IVM and can be imaged by two-photon excitation using near infrared laser wavelengths. We compared positive versus negative contrast imaging of the BM by co-injecting Hoechst 33342 and 70 kDa TRITC-dextran (Fig 3a). Hoechst labeling showed pronounced variation in cell brightness (Fig 3b), while the cell-impermeable labeling in the interstitium is subjected to less of cell-dependent variations (Fig 3c). Consequently, the negative contrast image (Fig 3c) has a more uniform appearance, and the crowded cellular microenvironment of the BM is readily apparent.

As an alternative to the Hoechst staining, a “universal” reporter mouse, such as the actin-GFP or the actin-dsRed mouse, can be used to provide positive contrast when cell type-specific expression of the FP is not needed [15]. We compared positive versus negative contrast imaging of the BM by intravenous injection of 10 kDa Rhodamine B-dextran into an actin-GFP mouse. While GFP+ cells were abundantly present throughout the BM (Fig 3d), pronounced, cell-dependent variations in the GFP’s brightness was once again observed, likely due to cell-type- or cell-cycle-dependent expression level of the FP. Moreover, the boundaries of individual cells were difficult to discern in regions of high cell density due to overlapping signals from adjacent cells.

To quantify our observations of heterogeneity in cell brightness, we plot the distribution of cell intensity values. Intensity of each cell was calculated by dividing the sum of its intensity values by its number of pixels. As shown in Fig 3f, the values corresponding to the Hoechst image (Fig 3a) show a broad distribution, ranging from a small fraction of cells at near saturation intensity to the majority of cells displaying low intensity. Similarly, the cell intensity values (Fig 3d) corresponding to the GFP image (Fig 3g) show a broad distribution of bright to dim GFP cells. In both cases, the distribution for the negative contrast images demonstrates more uniform cell brightness, as quantified by the Q3-Q1 interquartile range. In addition, the boundaries between individual cells are more clearly delineated in the negative contrast image, as illustrated by the line intensity profile shown in Fig 3h.

We also compared the photobleaching kinetics of actin-DsRed labeled cells and FITC-dextran labeled interstitium by injecting 70 kDa FITC-dextran into an actin-DsRed mouse and acquiring images while continuous scanning a 3D volume. As shown in Fig 3i, the interstitial FITC-dextran is less prone to photobleaching, as the dye is continuously replenished by the interstitial flow [8, 16]. This feature is particularly useful for extended time lapse imaging and long-term observation of cellular dynamics, as described below.

### Quantitative analysis of cell locations, density and sizes using negative contrast imaging

**Fig 4:**
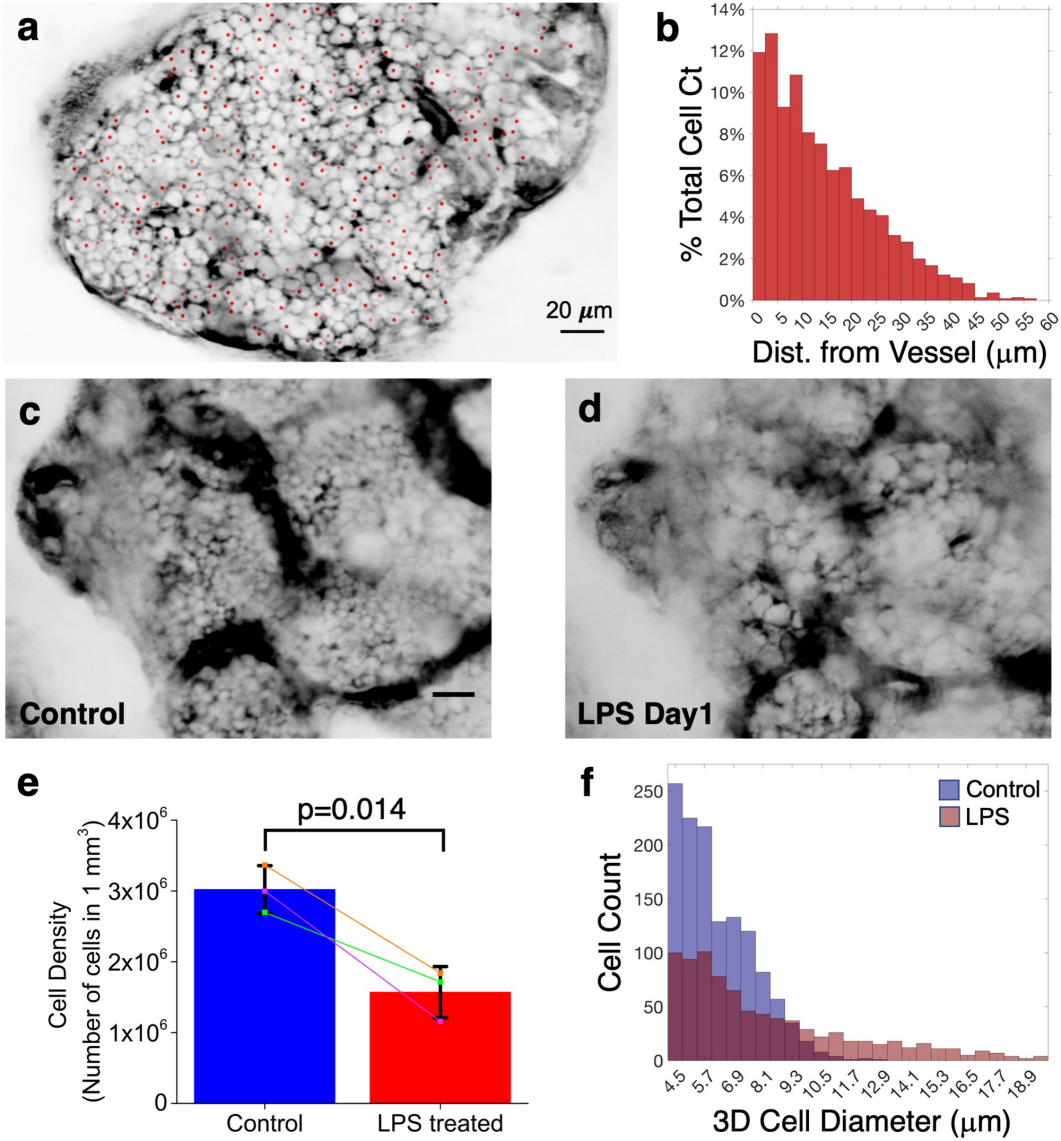
Quantitative analysis of cell locations, density and sizes using negative contrast imaging. **a**, Negative contrast image of a BM cavity, 10 μm below the endosteum. Centroid of each cell is marked as a red dot. The centroids of some cells are at other z-depths (full 3D stack is shown in S3 Movie). **b**, Histogram of the Euclidean distance between the cell centroids to their nearest blood vessel wall. ~90% of the cells are within 30 μm to the nearest blood vessel. N = 4 mice. **c-d**, Representative negative contrast image of the same BM cavity before and one day after LPS treatment. N = 3 mice (total of 18 z-stack locations) **e**, Comparison of cell density measured before and one day after LPS treatment (N = 2 mice, total of 3 z-stack locations). Significant decrease in cell density was observed after LPS treatment. Statistical analysis was performed by using paired sample *t* test (p value = 0.014). **f**, Histogram of cell sizes in a BM cavity before (blue) and one day after (red) LPS treatment. Scale bar for all images = 20 μm.

The vasculature and the endosteal surface are two major anatomic landmarks that are commonly used to track cell localization in the BM. The distribution of distances from individual cells to its nearest blood vessel or the endosteal surface is a useful metric to analyze cell localization [17–19]. By comparing the distance distribution of a specific cell population to a reference distribution, it is possible to assess whether the cell population of interest exhibits preferential localization relative to a random subset of BM cells. Currently, the most common method for generating the reference distribution is by artificially placing random dots in the BM during image analysis [20]. Negative contrast imaging circumvents the need to generate artificial dots by visualizing the actual distribution of BM cells and marking the position of each cell in 3D without requiring specialized fluorescent mice. S3 Movie shows a z-stack of negative contrast images acquired after the intravenous injection of 10 kDa FITC-dextran. The positions of individual BM cells are also depicted (red dots), defined by their centroid coordinates. Fig 4a shows a single optical section from the stack. After 3D segmentation of the vasculature (see Online Methods), the distance distribution of each cell to the nearest blood vessel wall was computed as shown in Fig 4b.

The 3D analysis described above also enables the determination of BM cell density, defined as the number of cells per volume, which can then be tracked over time by sequential imaging. Fig 4c and 4d and S4 Movie shows negative contrast images and the corresponding z-stack movie of the same BM cavity before (Fig 4c) and one day after (Fig 4d) administration of lipopolysaccharides (LPS) from *E*. *coli*, an agent known to trigger systemic inflammation and massive neutrophil egress from the BM [21]. As expected, a significant decrease in the cell density was observed in response to LPS (Fig 4e; also see Online Methods). In addition, quantitative analysis of cell diameters indicates that the number of cells with diameter less than 10 micrometer decreases significantly after LPS treatment (Fig 4f). This is supportive of the egress of smaller cells including neutrophils, as shown previously using the adoptive transfer of Ly6G^+^ neutrophils [21].

### Visualizing cell dynamics by negative contrast imaging

One of the most powerful features of IVM is the ability to track cell motion and quantify cellular dynamics by acquiring real-time or time-lapse movies. As mentioned above, negative contrast is suitable for extended time-lapse imaging because photobleaching is minimized. BM cells were visible by negative contrast for more than two hours after intravenous injection of 10 or 70 kDa dextrans. S5 Movie shows a single plane of a 3D time-lapse movie acquired at 1 z-stack per minute for a total of 104 minutes. Multiple types of cell motion can be observed in the BM, including cell rolling in sinusoidal blood vessels, transendothelial migration, and interstitial cell migration.

**Fig 5:**
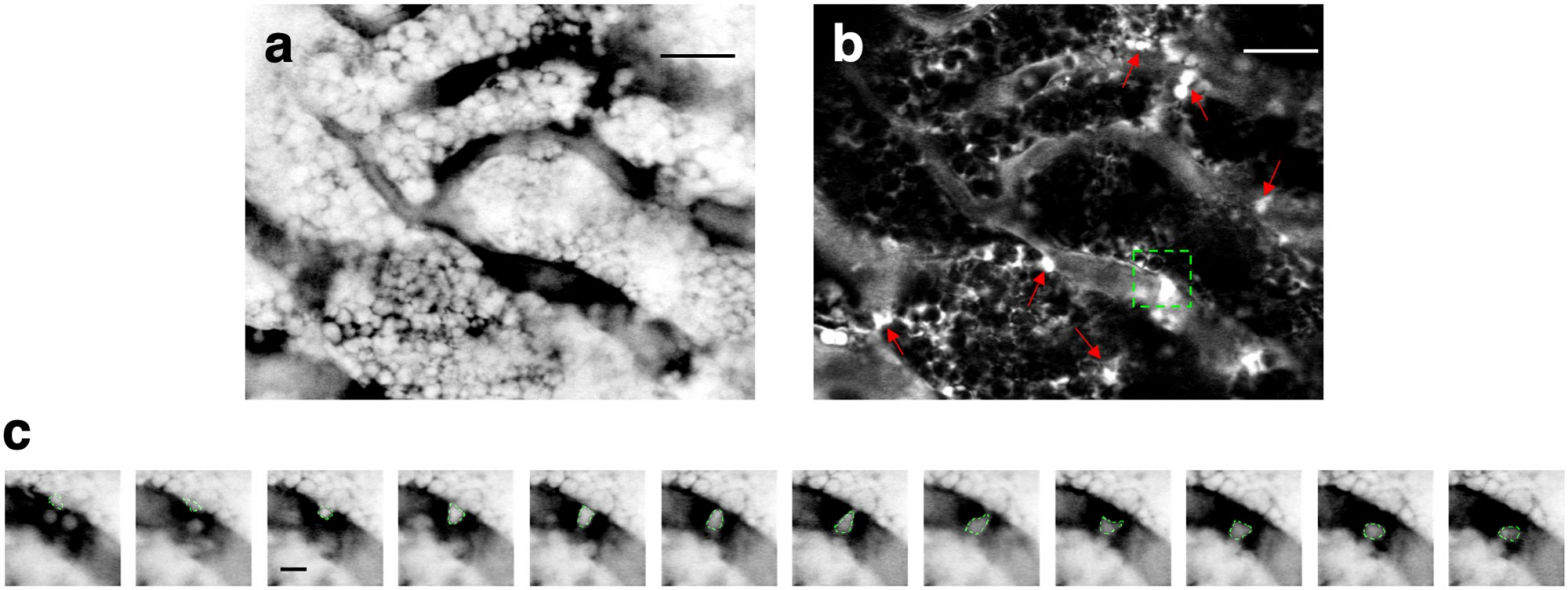
Visualizing BM cell dynamics by negative contrast imaging. **a**, A single snapshot from a time-lapse negative contrast imaging sequence of a mouse BM (the sequence is shown in S6 Movie). **b**, A standard deviation image created from the time lapse sequence. Bright regions with high standard deviation values are indicated by red arrows. These areas correspond to the regions of high cell motility. The dotted rectangle indicates the location of a transendothelial migration event. Scale bar = 30 μm. **c**, Time lapse negative contrast images of a cell transmigrating across the endothelial wall. The boundary of the cell is indicated by a green dotted line. Consecutive images were taken 30 seconds apart. Scale bar = 5 μm. Representative of N = 5 mice.

To analyze cell motion, we first performed a simple standard deviation analysis of the time lapse image sequence. Pixels with high standard deviation indicate regions where cells are moving, whereas pixels with low standard deviation indicate locations where cells are static (Fig 5a and 5b). The standard deviation image therefore highlights the regions to zoom in for analyzing cell motion in the corresponding movie. For example, by zooming into the green dotted rectangle in Fig 5b, we can pinpoint the high standard deviation in this region as resulting from transendothelial migration featured in Fig 5c and S6 Movie. In general, blood vessels have higher standard deviation values because of the intravascular cell motion, but the standard deviation image also identifies regions outside the blood vessels with robust cell motion (arrows in Fig 5b).

Examples of cell dynamics in the LN, visualized by negative contrast imaging, are shown in S7 and S8 Movies. Consistent with the immune surveillance activity of the SCS [22–24], more motile cells are observed in the SCS (S7 Movie) compared to the less motile B cells in the follicle (S8 Movie).

**Fig 6:**
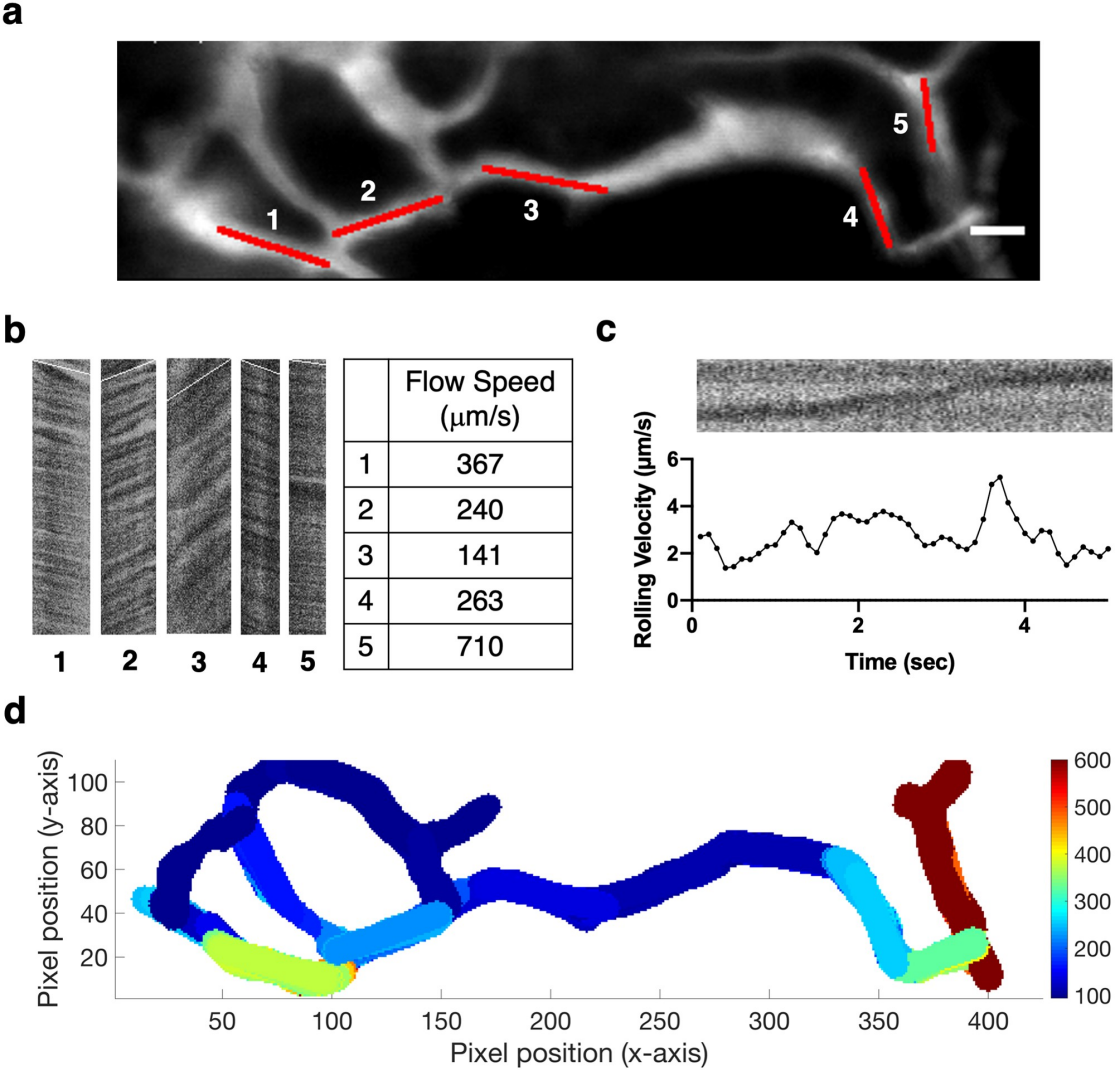
Automated blood flow profiling with negative contrast time series. **a**, The vascular network used for automated blood flow profiling, as an averaged image of the 120 fps, 2 s long negative contrast time series (S9 Movie). Scale bar = 20 μm. **b**, Digital line scan image for each red-marked vessel segment in **(a)**. Conceptually, each image is a vertical montage of the segment imaged at consecutive time points; a stationary cell would display as a vertical dark stripe. The slope of the stripes was estimated by Radon transform and overlaid on the images in white. Blood flow speeds were calculated from the slope value. **c**, Change in rolling lymphocyte velocity calculated over time. The raw digital line scan image shows the shadow of the rolling lymphocyte moving along the vessel (top). The velocity was calculated every 0.1 seconds at 30 fps. **d**, Flow profile of the 2D vascular network shown in **(a)**, generated by automated vessel segment selection and local flow speed mapping. Speed scale is in μm/s. N = 5 mice.

Cell motion in negative contrast images can also be used for blood flow measurements. A common method for measuring blood flow in IVM is by repeatedly scanning a line along a blood vessel and tracing the negative contrast of individual red blood cells (RBCs) over time. In this physical line scanning method, the blood flow velocity is measured one vessel at a time [25]. Here, we acquired 2D negative contrast images at high frame rates (up to 120 frames per second, S9 Movie) and performed digital line scanning on the time series image stack, which allowed us to obtain the flow profile of all blood vessels in a 2D vascular network in one shot. Fig 6a shows a 2D vascular image of the BM acquired after intravenous injection of 70 kDa FITC-dextran. Multiple vessel segments within the vascular network were selected for digital line-scanning (Fig 6b). The dark and bright stripes in the resulting images indicate the presence of dark cells and bright plasma, respectively. Blood flow speeds were calculated from the slopes of these stripes [26–28]. We used the same method to measure the velocity of rolling cells (Fig 6c). By automating the selection of vessel segments and mapping the local flow speeds, we were able to generate a comprehensive flow profile of a 2D vascular network shown in Fig 6d. At the current frame rate of 120 fps, our upper reportable speed limit is 1 mm/s, which is sufficient for the majority of sinusoidal blood vessels within the BM.

## Discussion

Negative contrast imaging overcomes a well-known limitation in standard fluorescence microscopy, the fact that one only sees what one labels. By highlighting all the extracellular spaces accessible to the dye, negative contrast imaging provides a complementary view, outlining cells that are not labeled. In complex tissues where it is often impractical to label all the cells *in vivo* using standard labeling approaches (or impossible without *a priori* knowledge of all the cell types present in the tissue), negative contrast offers a “one stop” approach to gaining an unbiased overview of the cellular context within which the labeled and unlabeled cell populations coexist. The technique has previously been used to guide whole-cell patch clamp recording of unlabeled neurons by local delivery of the dye into the brain parenchyma through a patch pipette [8]. Extending the technique to superresolution microscopy, exquisitely fine details of the neuropil have been revealed using *ex vivo* brain slices, the extracellular space of which is perfused with a fluorescent artificial cerebrospinal fluid [16]. Here we show that commonly used vascular and lymphatic tracers for IVM also provide negative contrast imaging of the BM and LN. The BM is endowed with a dense network of highly permeable sinusoidal blood vessels [9], which allows perfusion of fluorescent dextrans with molecular weight as high as 70 kDa in the extravascular space within minutes after intravascular injection (Fig 1 and S2 Fig). The extent of the negative contrast is expected to be impacted by disease. In particular, the vascular permeability is increased in many disease models (such as inflammation, radiation injury and chemotherapy), causing the dye to fill the interstitial space at even faster rates than the steady-state BM. Similar interstitial perfusion behavior is expected in other tissues with high vascular permeability, such as solid tumors [1]. The LN, on the other hand, is perfused by drainage of the lymphatic fluid, carrying the dye first to the sub-capsular sinus of the LN and then into the cortex. We observed dye penetration both along the system of specialized conduits as previously described [13, 14] and in the interstitial space, causing the cells in the cortex to become visible by negative contrast (Fig 2).

The method we describe in this work enables negative contrast imaging in a simple-to-implement form that can be used on an ad hoc basis. It works on a standard two-photon fluorescence microscope without modification and requires only a single fluorescence channel, leaving the other channels available for use with existing specific cell labeling strategies to supply positive contrast. As the method described here involves taking the signal that is already present in the interstitial space after routine labeling of the vasculature or the lymphatics with fluorescent tracers and turning the signal into a negative contrast image, the failure rate is no greater than the failure rate for tracer injection into the vasculature or the lymphatics in a routine IVM experiment. Compared with Hoechst counterstaining or the use of “universal” fluorescent reporter mice (Fig 3), negative contrast imaging provides a more uniform view of unlabeled cells while circumventing some of the known issues of positive cell labeling that cause significant cell-cell variation in brightness, such as cell-type or cell-cycle dependent dye uptake [29, 30], efflux [29–31], or levels of FP expression. Hoechst dyes, for example, are known to be effluxed by the ATP-binding cassette (ABC) multidrug transporters, and are used to define a “side population” of hematopoietic or leukemic cells based on their dye efflux properties [32].

The extracellular localization of the cell-impermeable dye for negative contrast imaging reduces cell toxicity, including phototoxicity [8] and dark toxicity associated with the DNA binding dye (Hoechst). It is also more resistant to photobleaching because the dye is continuously replenished by the interstitial flow [8, 16]. Cell borders are more readily visible (Fig 3h), as it is not required for the dye to interact with the plasma membrane to “draw” the border.

We showed that cellular dynamics ranging from seconds (cell rolling and transmigration) to days (mobilization after LPS treatment) are observable with this technique, and that changes in cell density can be longitudinally monitored in the same mouse and at the same location of the BM on separate days (Fig 4c and 4d), which is not possible using histologic bone sections or flow cytometry enumeration.

By defining the centroid of each cell, it is possible to track the cell position over time and determine the distribution of cell distances to anatomic landmarks such as the endosteum, vasculature, or other positively labeled structures. Here we show that, due to the high BM vascular density, all BM cells are closely associated with the vasculature, with ~90% of the cells located within 30 μm of the nearest blood vessel in 3D (Fig 4b). The result is consistent with a previous estimate for the calvarium [33] and also with the finding in long bones, where the average spacing between sinusoidal vessels is about 46 μm [20], implying that the majority of cells are less than half this distance (~23 μm) away from the nearest blood vessel.

The negative contrast technique described also provides a comprehensive picture of cell dynamics, including motions of individual cells and blood flow. We were able to distinguish different types of cell movement, including transendothelial migration (Fig 5c) and leukocyte rolling (Fig 6c). Using digital line scanning and a single time series image stack, we were able to extract blood flow speeds from all vessels within a 2D vascular network (Fig 6d). The method is readily extendable to 3D by repeating the steps at different optical sectioning depths, and is more efficient compared to physical line scanning that requires individual vessels to be scanned in sequence. We were also able to detect the changes in rolling velocity of leukocytes inside the vasculature (Fig 6c). While physical line scanning allows for higher reported speed limits, the range of measurable velocities permitted by our microscope setup is already sufficient for analyzing most of the sinusoidal blood vessels within the BM [25].

Consideration needs to be made regarding the choice of dye and route of injection. The foremost criterion is that the dye needs to be cell-impermeable. In addition, the dye retention in the interstitial space needs to be sufficiently long to allow adequate time for image acquisition. Small molecules such as AlexFluor 488 are cell impermeable, readily perfuses the interstitial space, and provides negative contrast, but the contrast disappears in <30 min as the dye is cleared rapidly. The retention time is increased when fluorophores are conjugated to 10–70 kDa dextrans which stay in the interstitial space for ~2 hours, about the length of our intravital imaging session. Finally, when used with positive cell labels, the spectral overlap of fluorophores needs to be minimized. We specifically chose orange/red fluorescent dyes due to their spectral compatibility with GFP labels, and we have used FITC-dextran when GFP was not present. Multiplexing with far red contrast agents can be more challenging due to the long red fluorescent tail of the orange-red fluorescent dextrans. Nevertheless, we have routinely multiplexed them with DiD, Cy5.5, and farther red contrast agents. Tail vein dye injection is a suitable alternative to retro-orbital dye injection. Both techniques have been used successfully for the delivery of dye and imaging agents, but retro-orbital injections are preferred for negative contrast imaging due to the high success rate, ease of use, and the ability to inject after the mouse has been mounted for imaging without any prior setup.

Like other imaging techniques, the negative contrast method has its limitations. The dextrans used in this study (10–70 kDa) remain in circulation for ~ 2hrs after intravenous administration, and act as the source for interstitial dye replenishment, but they remain subjected to normal clearance and need to be re-injected for longer imaging sessions. In addition, despite the dye being cell-impermeable, a small number of cells can take up the dye and reduce or abolish the contrast locally. Round-shaped cells are more readily visualized than flat cells with thin bodies or protrusions, such as endothelial cells, dendritic cells, and reticular cells. Improvement in spatial resolution will be key to imaging these finer structures [16]. The resolution for negative contrast imaging is not different from standard confocal or two-photon microscopy and is limited by how well the scanning laser beam can be focused (i.e., its point spread function). Objects smaller than the point spread function cannot be resolved but can nevertheless be detected, provided the objects are sufficiently bright. For negative contrast imaging, this means the extracellular space can be detected even if the width of this space is below the resolution limit, as long as there are enough dye molecules present in this space. It follows that cell borders can be delineated using negative contrast even when adjacent cells are closely packed, such as in the LN and the BM. Such densely packed cells are difficult to resolve using standard labeling techniques but can be distinguished by negative contrast (Fig 3h). On the other hand, a dark (unlabeled), thin cellular structure surrounded by bright extracellular fluid is difficult to detect, especially as the point spread function is degraded when imaging through bone and with increased imaging depth. Techniques for wavefront shaping, for example using adaptive optics [34–37], will be important for restoring the point spread function and improving the resolution.

In conclusion, the negative contrast method described here provides a means to generating a more comprehensive view of tissue beyond specifically labeled cell populations typically visualized by IVM. The only requirement for negative contrast imaging is perfusion of the interstitial space with a cell-impermeable fluorescent tracer [1, 2]. As interstitial fluid flow is increasingly recognized for its role in maintaining tissue homeostasis, this route of dye delivery can be further explored to facilitate intravital imaging and assessment of tissue integrity both in the steady state and under inflammatory or other disease conditions [38–40].

## Methods

### Animal models

All animal experiments were conducted in compliance with the institutional guidelines and approved by the Institutional Animal Care and Use Committee (IACUC) at Massachusetts General Hospital (IACUC approval 2007N000148) and the University of California Merced (IACUC approval AUP19-0001). Male or female C57BL/6 wild-type mice (Jackson Laboratories Stock No. 000664) at the age of 7–12 weeks were used for all studies except for the imaging of the actin-GFP and actin-DsRed mice. C57BL/6-Tg(CAG-EGFP)131Osb/LeySopJ and B6.Cg-Tg(CAG-DsRed*MST)1Nagy/J mice at the age of 8–12 weeks (The Jackson Laboratory) were used for the negative contrast imaging of the mice with fluorescent proteins.

### Preparation, dosage, route of administration of dyes

Stock solutions of FITC-dextran, TRITC-dextran, Rhodamine B-dextran or Texas Red-dextran of 10 or 70 kDa molecular weight (Thermo Fisher Scientific, Sigma Aldrich) were prepared in Dulbecco’s phosphate-buffered saline (DPBS; without calcium and magnesium) at the concentration of 50 mg/mL (10 kDa dextrans) or 10 mg/mL (70 kDa dextrans). Stock solution of Evans blue (Sigma Aldrich) was prepared in DPBS at 1% weight/volume concentration. Reagent source and stock numbers have been included in S1 Table.

Dosage and route of administration of dyes were as follows. For negative contrast imaging of the BM, 90 μL (10 kDa dextrans) or 150 μL (70 kDa dextrans) of the dextran stock solutions were injected intravenously. We injected ~0.6 mg of dextran per mouse without normalizing for mouse weight. Intravenous delivery of dextran dyes into the BM in Fig 1 was performed using a catheter installed in the mouse tail to monitor the delivery of the dyes into the BM from the time point of injection. All other intravenous injections were performed retro-orbitally. For negative contrast imaging of the LN, 20 μL of Evans blue (Fig 2a) or 70 kDa FITC-dextran stock solution (Fig 2d) was subcutaneously injected into the footpad. Afterwards, the popliteal lymph node of the mouse was exposed following previously published protocol [41]. The lymphatic vessel and the popliteal lymph node were subsequently identifiable by eye (for Evans blue) or by using blue (440–460 nm) flashlight and goggle with green (500–560 nm) light filter (for FITC-dextran). Blood vessels in the LN were labeled by intravenously injecting 100 μL Alexa Fluor 594 anti-mouse CD31 antibody solution (200 μg/mL in DPBS, clone: MEC13.3, BioLegend) 4 hours before imaging. Nuclear counterstaining in Fig 3a and 3b was accomplished by intravenous injection of Hoechst 33342 (10 mg/mL in DPBS, Thermo Fisher Scientific) at a dose of 10 mg/kg mouse body weight immediately before imaging. Systemic inflammation was triggered for Fig 4c–4f by intraperitoneal injection of lipopolysaccharides (LPS) from *Escherichia coli* (Sigma Aldrich) in DPBS vehicle at a dose of 0.8 mg/kg mouse body weight, at 24 hours before imaging.

### Intravital microscopy (IVM)

All negative contrast images were acquired using a custom-built multiphoton/confocal microscope as described in detail in previous reports [42, 43]. Briefly, our microscopes are custom-built laser-scanning multiphoton/confocal microscopes constructed on a combination polygon (fast axis) and galvanometer (slow axis) scan engine. A total of six photomultiplier tubes provide three detection channels in the confocal (descanned) beam path and three two-photon channels in the non-descanned beam path. Full field of view 8-bit RGB images are acquired at 30 frames per second (fps) with 500 x 1000 pixels or up to 120 fps with 125 x 1000 pixels by selecting input from any combination of the six detectors. Although we build our own microscopes, negative contrast microscopy does not require a custom multiphoton system and is compatible with most commercially available multiphoton imaging systems. A skull skin flap was created to optically access the calvarial BM in vivo. The mouse head was stabilized in a custom-built mouse restrainer and maintained in anesthesia using 1.25% vaporized isoflurane. For LN imaging, the popliteal LN was exposed following the previously published protocol [41]. A femtosecond Ti:Sapphire pulsed laser with variable wavelength of 700–1000 nm was used as two photon excitation and second harmonic generation source. The wavelength of the excitation was chosen to efficiently generate two photon excitation fluorescence from multiple fluorescent probes simultaneously: 775–800 nm for FITC-dextran, Evans blue and Alexa-594 (Fig 2), 780 nm for Hoechst 33342 and TRITC (Fig 3a), 840 nm for EGFP and Rhodamine B-dextran (Fig 3d), and 800, 840, 900 or 960 nm for FITC-dextran. A polygon scanner with 17280 lines/s scan rate enabled video rate imaging at 30 fps. For Fig 6a, the frame size was adjusted to achieve a frame rate of 120 fps. A water immersion 63x objective lens (NA = 1.15) was used for all studies. The laser power was about 70 mW at the sample. Fluorescence emission was collected by photomultiplier tubes with proper dichroic and filter settings corresponding to the fluorophores of interest. Specific bandwidths of the filters used for the detection of Hoechst 33342 and FITC were 420–500 and 500–550 nm, respectively. For the detection of TRITC, Rhodamine B and Texas Red, filters with spectral width of 550690 nm were used. The second harmonic generation signal from collagen was collected to visualize the skull calvarial bone and capsule of the lymph node.

### Quantitative analysis of BM cell locations, density and sizes

MATLAB codes developed in-house were used for the analysis (downloadable at https://github.com/juwellwwu/NCCENTROID3D). The algorithm used for counting and positioning of cell centroids was based on watershed transformation. Prior to the segmentation, the vascular signal from the starting image stack was removed, and the remaining interstitial signal cleaned up. Background signal was also measured. Marker-controlled watershed segmentation was then performed, which provided the information and cell centroid position and cell size in 2D. Bone, vessel and low signal regions were excluded by masks. The 2D cell information was then used to build a 3D model of cell packing, after the assumption that most marrow cells are roughly spherical. 3D cell centroid counts and positions were finally drawn from the model.

For quantitative comparison of cell density pre- and post-LPS experiment (Fig 4c–4e), individual cells were recognized by three-dimensional image segmentation from the negative contrast image stack of the bone marrow with the size of 100 x 100 x 20 (μm^3^). Three-dimensional segmentation to recognize individual cells was performed either by using inhouse MATLAB code and ImageJ plugin, ‘MorphoLibJ’ [44]. Images at the same location before and after LPS treatment were selected by using the structure of the bone and the blood vessels as references. One negative contrast image per mouse (total 3 mice) was used to see the effect of LPS treatment.

### Automated blood flow profiling with negative contrast time series

MATLAB codes developed in-house were used for the analysis (downloadable at https://github.com/juwellwwu/NCFLOWSPEED). Briefly, the time sequence of negative contrast images was averaged and the vascular network was identified by image segmentation. Vessel segments were selected by Hough transform after skeletonizing the segmented image. For each vessel segment, the slope of the stripes in its digital line-scan image was calculated using previously published algorithms [26–28]. The sign of velocity values indicates the direction of flow. Digital line-scan images can also be generated using the “Dynamic Reslice” function in ImageJ.

### Histomorphometric examinations

After euthanasia of mice, the femurs were dissected. Samples were fixed in 4% paraformaldehyde, decalcified in 20% ethylenediaminetetraacetic acid (EDTA) at 4 °C for 2 weeks, and then embedded in paraffin. Longitudinal 5 μm serial sections were sliced and subjected to hematoxylin and eosin (H&E) staining.

### Statistics

Boxplots in Fig 3f and 3g were drawn using the ggplot package in R. The box length shows the interquartile range (IQR; 75th percentile (Q3) − 25th percentile (Q1)) of the data. The line inside the box indicates the median (50th percentile). Whiskers are defined Tukey style, extending 1.5 x IQR from the box edges. For Fig 4e, statistical analysis was performed using a paired sample *t* test.

## Supporting information

S1 Fig Diagram of the negative contrast imaging technique. **a**, In standard fluorescence microscopy, positive contrast is used to label vasculature (red bar) and subsets of cells (green circles). **b**, In fluorescence microscopy with negative contrast, the interstitial space is labeled with fluorescent tracers via intravascular delivery or lymphatic drainage to visualize unlabeled cells (white circles) with negative contrast in the bone marrow (BM) and/or lymph nodes (LN).
(TIFF)

S2 Fig Two-photon negative contrast imaging of a mouse BM cavity, at different time points after the injection of a vascular dye. **Color panels**, Images taken at 3 s, 30 s, and 1 min after tail-vein injection of 70 kDa FITC-dextran, respectively, in a different mouse than Fig 1. **Grayscale panel**, The LUT of the 1 min image was digitally inverted such that cells appeared as bright objects above the dark interstitial background. Representative example from N = 3 mice.
(TIFF)

S3 Fig Brightfield image of histological section of mouse bone marrow. Grayscale image of the metaphysis of a mouse femur section (5 μm thickness) stained with hematoxylin and eosin. N = 1 mouse.
(TIFF)

S1 Table Reagent source and stock numbers. (DOCX)

S1 Movie Initial filling of the BM vasculature by Texas Red-dextran from the same mouse used for Fig 1. This video was acquired between 1 and 21 seconds after the tail vein injection of Texas Red-dextran, at a frame rate of 30 fps. Image size: 340 x 170 μm.
(MP4)

S2 Movie Initial filling of the BM vasculature by FITC-dextran from the same mouse used for S2 Fig. This video was acquired for the first 1 minute after the tail vein injection of FITC-dextran, at a frame rate of 30 fps. Image size: 340 x 170 μm.
(MOV)

S3 Movie Automated 3D cell centroid analysis in the BM. This video shows a negative contrast image z-stack of the BM. The Z-step is 0.34 μm. The 3D centroid of each BM cell is marked with a red dot. Scale bar = 20 μm.
(AVI)

S4 Movie The BM before and one day after LPS treatment. This video shows a negative contrast image z-stack of the BM before and one day after LPS treatment. The z-step is 0.34 μm. The 3D centroid of each BM cell is marked with a red dot. Scale bar = 20 μm.
(AVI)

S5 Movie BM cell dynamics observed in negative contrast imaging. This time lapse video of a single plane in the BM was acquired as a 3D time-lapse movie, at 1 z-stack per minute for a total of 104 minutes. Multiple types of cell motion can be observed, including cell rolling, transendothelial migration, and interstitial cell migration. Photobleaching of the dye in the interstitium was minimal. Image size: 150 x 150 μm. Representative of N = 5 mice.
(AVI)

S6 Movie Intravasation of a BM cell. This time lapse video shows the transmigration of a BM cell from the marrow space into a blood vessel (intravasation). Red arrow indicates the region where intravasation occurred. The video was acquired at 30 seconds per frame for a total of 10 minutes. Image size: 105 x 82 μm.
(AVI)

S7 Movie Cell motion in the popliteal lymph node near the subcapsular sinus (SCS). This time lapse video was acquired at 1 minute per frame for a total of 30 minutes. Image size: 390 x 186 μm. N = 1 mouse.
(AVI)

S8 Movie Cell motion in the popliteal lymph node near B cell regions. This time lapse video was acquired at 1 minute per frame for a total of 30 minutes. Cells are less motile compared to those in the SCS (S7 Movie). Image size: 390 x 186 μm.
(AVI)

S9 Movie Flowing red blood cells (RBCs) provide negative contrast in the BM vasculature. This video of the BM vascular network was acquired at a single plane, at 120 fps for a total of 2 seconds. Image size: 327 x 85 μm.
(AVI)
