# Supplementary figures and images for: Intravital fluorescence microscopy with negative contrast
Source: PLoS One. 2021 Aug 5;16(8):e0255204. doi: 10.1371/journal.pone.0255204 (PMC8341626; doi:10.1371/journal.pone.0255204)

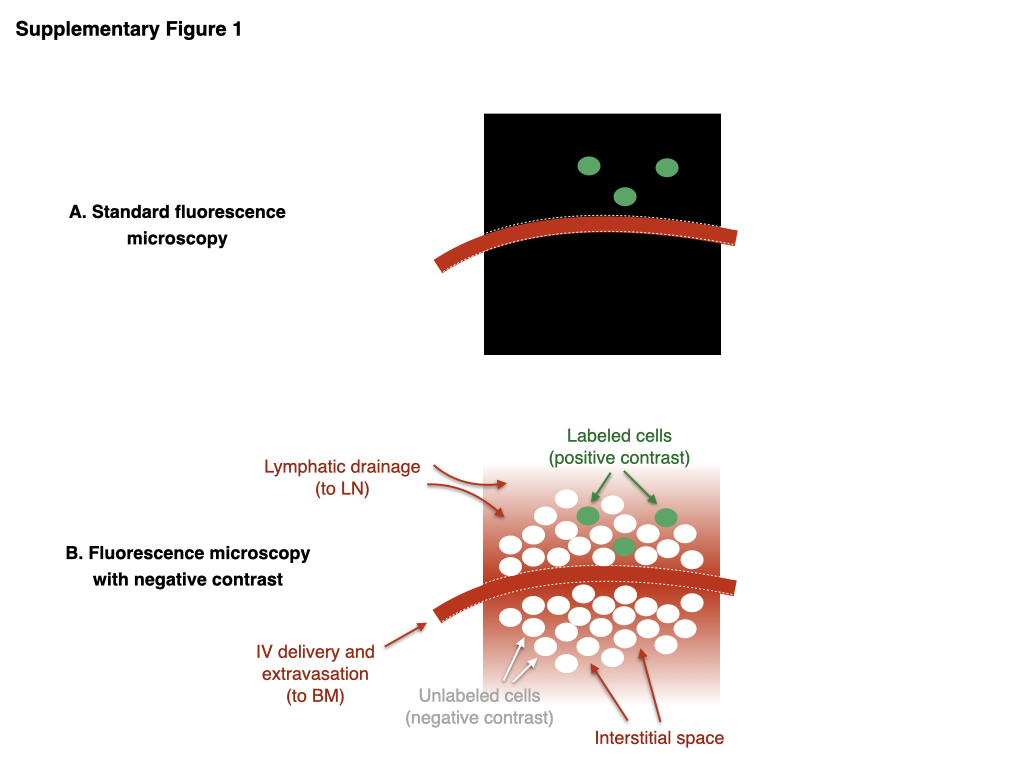

Supplement: S1 Fig — a, In standard fluorescence microscopy, positive contrast is used to label vasculature (red bar) and subsets of cells (green circles). b, In fluorescence microscopy with negative contrast, the interstitial space is labeled with fluorescent tracers via intravascular delivery or lymphatic drainage to visualize unlabeled cells (white circles) with negative contrast in the bone marrow (BM) and/or lymph nodes (LN). (TIFF) [file pone.0255204.s001.tiff]

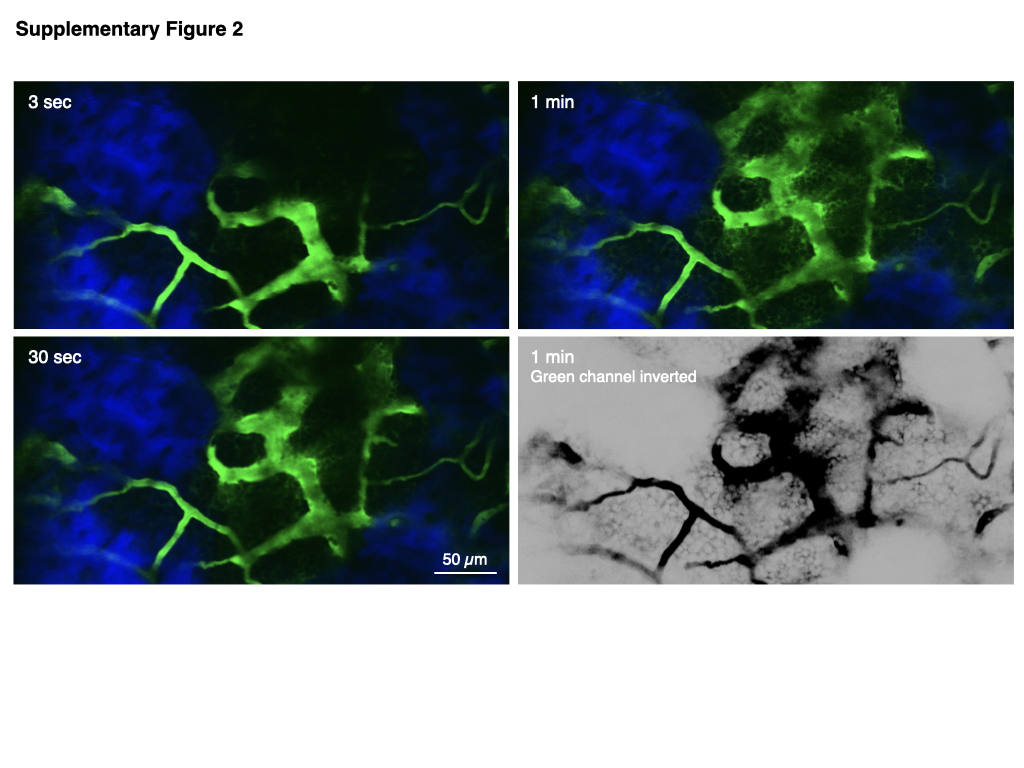

Supplement: S2 Fig — Color panels, Images taken at 3 s, 30 s, and 1 min after tail-vein injection of 70 kDa FITC-dextran, respectively, in a different mouse than Fig 1. Grayscale panel, The LUT of the 1 min image was digitally inverted such that cells appeared as bright objects above the dark interstitial background. Representative example from N = 3 mice. (TIFF) [file pone.0255204.s002.tiff]

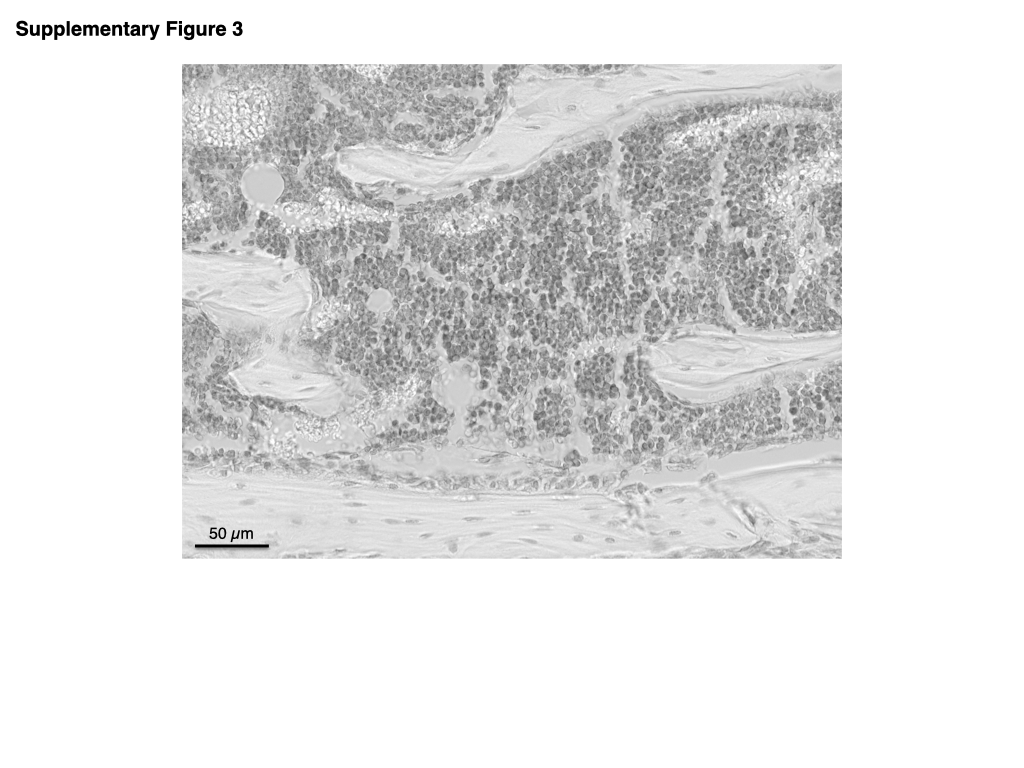

Supplement: S3 Fig — Grayscale image of the metaphysis of a mouse femur section (5 μm thickness) stained with hematoxylin and eosin. N = 1 mouse. (TIFF) [file pone.0255204.s003.tiff]
