# Supplementary material for: Intravital fluorescence microscopy with negative contrast
Source: PLoS One. 2021 Aug 5;16(8):e0255204. doi: 10.1371/journal.pone.0255204 (PMC8341626; doi:10.1371/journal.pone.0255204)
Supplement: S1 Table — (DOCX) [file pone.0255204.s004.docx]

**S1 Table**. **Reagent source and stock numbers.**

| *Reagent* | *Catalog Number* |
| --- | --- |
| *FITC-dextran* | *10k: Thermo Fisher Scientific #D1821 70k: Thermo Fisher Scientific #D1823 10k: Sigma-Aldrich #FD10S 70k: Sigma-Aldrich #46945* |
| *TRITC-dextran* | *10k: Thermo Fisher Scientific #D1816 70k: Thermo Fisher Scientific #D1819* |
| *Rhodamine B-dextran* | *10k: Thermo Fisher Scientific #D1824 70k: Thermo Fisher Scientific #D1841 10k: Sigma-Aldrich #R8881 70k: Sigma-Aldrich #R9379* |
| *Texas Red-dextran* | *10k: Thermo Fisher Scientific #D1828 70k: Thermo Fisher Scientific #D1830* |
| *Evans blue* | *Sigma-Aldrich # E2129* |
| *AlexaFluor 594 anti-CD31 solution* | *Biolegend #102432* |
| *Hoechst 33342* | *Thermo Fisher Scientific #H3570* |
| *Lipopolysaccharides from Escherichia coli* | *Sigma-Aldrich #L2143* |
